# The Trade-Off between Dietary Salt and Cardiovascular Disease; A Role for Na/K-ATPase Signaling?

**DOI:** 10.3389/fendo.2014.00097

**Published:** 2014-07-17

**Authors:** Joe X. Xie, Anna Pearl Shapiro, Joseph Isaac Shapiro

**Affiliations:** ^1^Department of Medicine, University of Colorado School of Medicine, Aurora, CO, USA; ^2^Department of Medicine, University of Toledo College of Medicine, Toledo, OH, USA; ^3^Department of Medicine, Joan C. Edwards School of Medicine, Marshall University, Huntington, WV, USA

**Keywords:** cardiotonic steroids, digitalis-like factors, fibrosis, sodium pump, signaling, renal failure, hypertension

## Abstract

It has been postulated for some time that endogenous digitalis-like substances, also called cardiotonic steroids (CTS), exist, and that these substances are involved in sodium handling. Within the past 20 years, these substances have been unequivocally identified and measurements of circulating and tissue concentrations have been made. More recently, it has been identified that CTS also mediate signal transduction through the Na/K-ATPase, and consequently been implicated in profibrotic pathways. This review will discuss the mechanism of CTS in renal sodium handling and a potential “trade-off” effect from their role in inducing tissue fibrosis.

## Introduction

Increased dietary sodium chloride (NaCl) intake has been implicated in cardiovascular and renal diseases for some time (1), and this implication has recently become fairly solid (2). This relationship between dietary sodium intake and cardiovascular disease is demonstrated in several large scale studies, such as the international study of salt and blood pressure (INTERSALT) (3) and the dietary approaches to stop hypertension (DASH) (4). With this relationship so demonstrated, understanding the specific mechanisms underlying the deleterious effects of NaCl becomes timely and relevant to clinical management.

This review will focus on one of the factors linking dietary NaCl to cardiovascular and renal disease. We will specifically discuss the role of digitalis-like factors, also known as endogenous cardiotonic steroids (CTS), which function as innate inhibitors of the Na/K-ATPase (5). Although the existence of these endogenous factors has been controversial (6–8), this is no longer the case. Some of these recent breakthroughs include the chemical identification of specific CTS in experimental animals and humans (9, 10), establishment of normal and pathological concentrations for these substances as well as defining possible roles for CTS in animal models of and human disease states (11–13). We would also stress that the discovery of the cell signaling functions of the Na/K-ATPase and its role in molecular cellular biology (14–16) has also been quite relevant to this field. Here, we will emphasize the role of trade-off with respect to CTS signaling and Na homeostasis.

## Renal Salt Reabsorption and the Evidence for “Third Factor”

The microscopic architecture of the kidney involves the attachment of vascular filtering units called glomeruli with tubules that modulate the quantity, electrolytes, and acid-base content of tubular fluid, which ultimately becomes urine. Simplistically, the tubules can be roughly broken down into proximal, where 60–80% of all Na and water reabsorption occur and distal, the nephron segments responsible for the fine tuning of what is excreted as urine.

Clearly the renin–angiotensin–aldosterone system, vasopressin and the sympathetic nervous system are critically important in mammalian volume regulation as well as to the maintenance of blood pressure in the face of a hypovolemic insult (17). However, it is very clear that perturbations in these systems cannot explain natriuretic responses to acute or chronic expansion of blood volume (18). This point was first demonstrated in 1961 in a classic paper by de Wardener and colleagues (19). This study showed that natriuresis induced by saline infusion occurred even if renal perfusion pressure and glomerular filtration rate (GFR, factor 1) and aldosterone concentrations (factor 2) were prevented from changing. This so called “third factor,” which we now understand is (are) CTS, was a “hot” topic in the 1960s and 1970s, and was even incorporated into Guyton’s model for circulatory homeostasis (20). Cort and Lichardus observed that a circulating substance in animals subjected to carotid artery occlusion induced natriuresis in different mammals and inhibited sodium transport in frog skin (21). Buckalew showed that an ultrafiltrate of volume-expanded dogs inhibited sodium transport in toad bladders. They went on to propose that the active substance was an inhibitor of the Na/K-ATPase (22). Gonick and coworkers showed that volume expansion in rats, in fact, produced a chemical which did inhibit the ATPase activity of rat kidneys (11). In 1980, Gruber and Buckalew noted that elevated levels of circulating digoxin-like material was seen in volume-expanded dogs (23). Other important contributions were made in the laboratory of Schrier and de Wardener over the next decade (24–26). However, doubt as to the validity of Na/K-ATPase inhibitors developed during the 1980s and 1990s because of inconsistencies in the reported results. In particular, prevailing CTS assays were based on cross-reactivity of CTS with antibodies to digoxin. This cross-reactivity of the commercially employed anti-digoxin antibodies to CTS varied considerably (27–32). Probably, the most important inconsistency was that digitalis did not appear to be natriuretic in normal subjects (33). On this background, atrial (and brain) natriuretic peptide(s) were discovered, were obviously natriuretic, and their concentrations (which could be easily measured) were increased in volume-expanded states (34–38). Undoubtedly, these points deflected interest from the study of CTS. However, enthusiasm was renewed in the recent past for the following reasons. First, several CTS have been isolated from experimental animals and humans and chemically characterized. Specifically, marinobufagenin (MBG) as well as telecinobufagin (TCB) have been isolated from plasma and urine (9). Ouabain has also been identified although there is still some debate as to whether this is ouabain or something distinct, which also reacts to anti-ouabain antibodies (10, 39). The concentrations of ouabain (or ouabain like compound) and MBG appear to be in the range of 200–2700/min in humans, depending on whether disease is present (5, 40, 41). Plasma levels of TCB and bufalin are less well defined at present. Also, quite importantly, a signal cascade has been identified, which does not appear to involve enzymatic inhibition of the Na/K-ATPase. This signaling pathway involves CTS binding of the caveolar Na/K-ATPase in the company of Src and the EGFR and the elaboration of a signal cascade, which involves the generation of reactive oxygen species (ROS) (14, 16). Both of these concepts have been extensively reviewed (42–44).

## “Trade-Off” Concept, a Historical Perspective

The concept of “trade-off” plays an extremely powerful role in physiology. This is perhaps best described by Neal Bricker who postulated that in renal disease, the hormonal forces driving nephrons to maintain fluid and electrolyte homeostasis would be complicated by the untoward consequences of these elevated hormones mediating other effects, essentially creating the signs, symptoms, and pathophysiologic changes associated with the uremic syndrome (45, 46). As sodium (Na) handling is so critical to volume balance, electrolyte homeostasis, and acid-base status, it is not surprising that Bricker formulated this hypothesis to involve the Na/K-ATPase.

**Figure 1:**
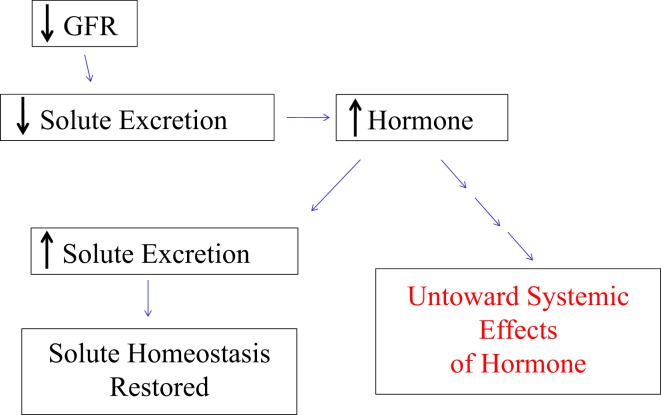
**The schematic in figure demonstrates Bricker’s proposed trade-off mechanism by which physiologic changes such as reduced glomerular filtration rate (GFR) leading to the increased generation of a hormone can produce the desired effect in solute homeostasis, but with untoward effects in renal and other tissues**.

Bricker speculated that an inhibitor of the Na/K-ATPase would circulate in increased concentration as a response to decreased GFR in order to maintain Na homeostasis (45). This inhibition would subsequently lead to decreased renal Na reabsorption, hence the maintenance of Na homeostasis (Figure 1). Unintended effects of higher concentrations of this Na/K-ATPase inhibitor would be responsible for some of the symptoms, signs, and abnormal laboratory results seen with chronic renal failure as well as potentially contribute to the progressive nature of chronic kidney disease (45, 47–50). As we will detail in this review, a potential consequence of increases in natriuretic hormone levels, specifically elevated CTS levels may be the profibrotic effects of these molecules (51). Before we address this, however, it may be useful to briefly discuss the evolution of our understanding of the Na/K-ATPase (45, 46), which had been described and characterized several decades before (52).

## Discovery of the Na/K-ATPase, Its Role in Signaling Cascades vs. Ion Transportation

The Na/K-ATPase was discovered by Skou in 1957 (53). This protein was demonstrated to be responsible for the electrogenic exchange of sodium and potassium (54). The Na/K-ATPase, also called the sodium pump, is present in all living cells (55). Although there has been some evolutionary modification of the sodium pump, in all multicellular animal cells, the sodium pump consists of (at least) a dimer of an alpha and beta subunit and is considered a member of P-type ATPases (43). Different isoforms of the alpha and beta subunits have been identified and are believed to have functional differences, a topic which has been extensively reviewed (56). Genes encoding the alpha-1 and alpha-2 isoforms reside on the chromosome 1 whereas alpha-3 appears to be coded for on chromosome 19 and alpha-4 (present only in sperm) is mapped to chromosome 13 in humans (57). The act of pumping sodium and potassium is accompanied by changes in conformation and phosphorylation state (43). It also requires energy provided by the hydrolysis of ATP as was initially identified also by Skou (58).The work of Skou was ultimately matured into the currently accepted Post-Albers model for Na/K-ATPase pumping function (43). The alpha 1 subunit of the Na/K-ATPase has 11 transmembrane domains as well as several well defined cytosolic regions referred to as the N, P, catalytic, and A domains (43). Interestingly, the development and maintenance on an evolutionary scale of caveolin and Src binding motifs, which are scattered throughout these cytosolic domains appeared to occur between single celled animal structures and slime mold (59).

**Figure 2:**
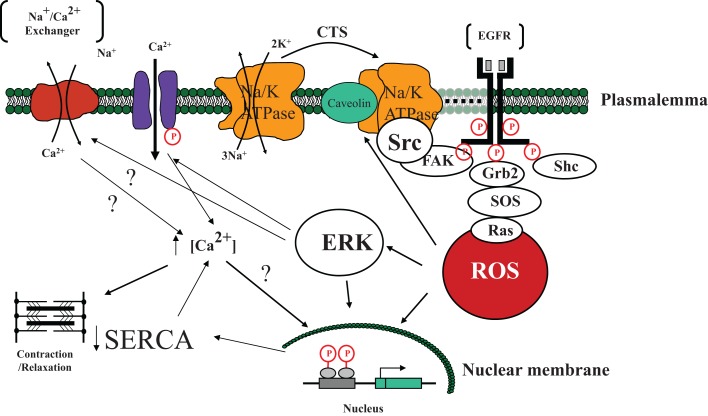
**A schematic illustrating the involvement of cardiotonic steroid (CTS) – induced Na/K-ATPase signal cascade initiated by the Na/K-ATPase mediated activation of Src tyrosine kinase and subsequent downstream targets eventually leading to the development of reactive oxygen species (ROS)**. Specifically, we postulate that in the microdomain of caveolae, the Na/K-ATPase functions as a scaffolding protein, interacting with CTS and changing conformation so as to active Src. Src then trans-activates the EGFR which leads to a signal cascade involving FAK, Shc, Grb2, and SOS resulting in the generation of ROS which in turn activates additional Na/K-ATPase molecules as well as causes downstream activation of ERK as well as effects on the nuclear transcription (43). ERK activation has effects on both L-type channels and possibly the Na/Ca exchanger with net effect to increase cytosolic Ca in some tissues (15). Nuclear effects in myocardial tissue include downregulation of SERCA transcription and translation (70). Abbreviations: EGFR, epidermal growth factor receptor; FAK, focal adhesion kinase; Shc, Src homology-2 domain containing protein; Grb2, growth factor receptor-bound protein-2; SOS, son of sevenless protein; ERK, extracellular-signal-regulated kinase; SERCA, sarcoplasmic/endoplasmic reticulum calcium ATPase.

In the late 1990s, the laboratory of Dr. Zijian Xie added a significant wrinkle to this understanding. While it is certainly possible that some signaling does occur through the chemical inhibition of the plasmalemmal Na/K-ATPase, it does appear that other mechanisms must be proposed to explain the signaling. In fact, it appears that the specific Na/K-ATPase molecules responsible for the greatest amount of signaling in response to the binding of CTS are actually not involved in pumping sodium or potassium (60). In the late 1990s, Dr. Xie and colleagues observed that in neonatal cardiac myocytes, ouabain caused increases in ROS measured with CMDCF (14). It was further noted that some of the downstream effects of ouabain were blocked by *N*-acetyl cysteine (NAC) or vitamin E. These increases in ROS could be demonstrated even when cytosolic calcium was maintained low by removal of extracellular calcium (16). It was further noted that Ras activation appeared to be necessary to see increases in ROS (16). Other studies determined that interactions between the Na/K-ATPase and Src appeared to initiate the signal cascade. The alpha 1 subunit of the Na/K-ATPase binds Src and appears to maintain it in an inactive state. However, binding a CTS appears to alter the Na/K-ATPase structure allowing Src to became activated which, in turn, trans-activates the EGFR, and begins the signal cascade which causes increases in ROS (61–64). The Na/K-ATPase–Src complex appears to function similar to a receptor tyrosine kinase. Downstream activation of PLC, PI(3)K, and PKC has also been established (15, 65–68) (Figure 2). The role of ROS in pump signaling has been extensively reviewed elsewhere (14, 16, 51, 69).

Although inhibition of the Na/K-ATPase is certainly one possible mechanism by which digitalis and related molecules might “signal,” it is important to emphasize that even transporting epithelia typically have a redundancy of Na/K-ATPase pumping units given that cytosolic Na levels live within a range ideally suited to regulate Na/K-ATPase activity. While it is possible that certain compartments of the cell see higher local concentrations of Na with modest inhibition of Na/K-ATPase pump activity, we emphasize that physiological and even pharmacological concentrations of digitalis do not demonstrably increase cytosolic Na concentrations in physiologically relevant preparations (42). We would further point out that most studies, including those from our lab, which demonstrate inhibition of the Na/K-ATPase by circulating substances do so with strategies to control for the cytosolic Na concentration (71–74).

Approximately one decade ago, a further analogy of Na/K-ATPase signaling to the signaling of receptor tyrosine kinases was established with the observation that CTS binding to the Na/K-ATPase in renal tissues triggers endocytosis of the CTS-Na/K-ATPase complex (75). Subsequent studies have demonstrated that this internalization is associated with endosomal accumulation of the Na/K-ATPase and its caveolar signaling partners, and that the process requires both caveolin (and caveolar structure) and clathrin (76, 77). We have gone on to demonstrate that this process appears to also regulate the expression of the apical sodium transporter, NHE3, as well as impact renal salt excretion *in vivo* (78–80). Recent data from the laboratory of Dr. Lingrel utilizing novel genetic manipulations of the different alpha 1 isoforms in mice indicate that it is the alpha 1 subunit, which can be considered the functional receptor for these CTS. Interestingly, the amount of Na/K-ATPase alpha 1 subunit as well as it is affinity for CTS appear to both positively correlate with the magnitude of the signaling effect (81–84).

Recently, we have made several observations that bring the consideration of ROS in the context of Na pump signaling in a new light. First, we found that the Dahl salt-resistant (R) strain of rats had a natriuretic response to a high salt diet, which did not require substantial increases in blood pressure (hence the term “salt resistant”) and was accompanied by activation of Src and ERK as well as redistribution in the renal proximal tubule cells of the basolateral Na/K-ATPase and apical NHE3. This was previously observed with the wild type Sprague Dawley animals (which were used as a founder population to generate Dahl R and salt sensitive, S, rats). In contrast, the Dahl S rats did not have this redistribution. Isolated proximal tubules from young Dahl R and S rats maintained on a low salt diet demonstrated ouabain sensitivity and insensitivity, respectfully, in terms of Src and ERK activation as well as redistribution of the NaK-ATPase and NHE3 (85). Moving back to LLC-PK1 cells, we noted that the signaling observed with ouabain or other CTS could be duplicated by exposure to an ROS generation system (Glucose Oxidase + Glucose), blocked by anti-oxidants (e.g., *N*-acetyl cysteine) and was accompanied by specific carbonylation of two amino acids in the A domain portion of the alpha 1 subunit (86). Given that the proximal tubules of Dahl S rats demonstrate considerable carbonylation of plasma proteins including the Na/K-ATPase prior to exposure to high salt *in vivo* or ouabain *in vitro* (unpublished data), this suggests that chronic oxidation of the Na/K-ATPase may lead to impaired signal transduction in the proximal tubule and a form of oxidant “fatigue.” Perhaps of even greater importance, the protein oxidation seen with both ouabain and glucose oxidase/glucose was found to be reversible in a biochemical rather than a physiological sense since removing ouabain or glucose oxidase/glucose led to the return to non-carbonylated proteins regardless of whether new protein synthesis or protein degradation were inhibited. In addition, signaling through the Na/K-ATPase appeared to impact the amount and degree of protein carbonylation induced by glucose oxidase/glucose suggesting a role for the Na/K-ATPase as both a receptor and amplifier of ROS (86). We had seen *in vivo* data supporting this concept in earlier studies discussed below. Although a feed-forward system (which this appears to be) suggests ongoing amplification, it seems clear that endocytosis of this molecular machinery would be an effective termination mechanism (87). Whether the oxidatively modified Na/K-ATPase is a trigger for endocytosis is a topic we are actively investigating at present.

On this background, it is useful to consider whether a CTS is effectively natriuretic *in vivo*. This discussion began many years ago regarding the CTS pharmacological agent, digoxin, or digitalis, which was noted to effect natriuresis in patients with congestive heart failure but not normal subjects (88). Currently, there remains debate as to whether a CTS such as ouabain is, in fact, natriuretic (89). Although clearly this is important in understanding the physiological relevance of the molecular mechanisms described above, we would caution the reader that the answer to this question may be different depending on the physiological state of the experimental animal or subject at the time of the study (80, 85, 90). That said, we would certainly concede that a correlation between renal Na/K-ATPase signaling or inhibition and natriuresis may not always be present.

## Role in Cardiac and Renal Fibrosis with Experimental Renal Failure

Concern that CTS signaling through the Na/K-ATPase might be profibrotic grew from several studies. First, we observed that experimental renal failure produced cardiac fibrosis in both rat and mouse (91). We would stress that human uremic cardiomyopathy is believed to also be complicated by fibrosis. When we performed active immunization prior to induction of experimental renal failure, the cardiac fibrosis was markedly attenuated. In a separate group of animals, infusion of MBG designed to achieve similar plasma levels of MBG as seen with experimental renal failure also caused cardiac fibrosis. Evidence for Na/K-ATPase signaling (e.g., Src and ERK activation) was see in both animals subjected to experimental renal failure or MBG infusion whereas active immunization against the MBG-Albumin conjugate attenuated this in the experimental renal failure group (51, 70, 91, 92). In addition, blockade of Na/K-ATPase signaling with active (or passive) immunization as well as pharmacologic blockade (see below) dramatically attenuated the oxidant stress in tissues seen with experimental renal failure (51, 91, 93, 94). Based on these animal studies, we next examined how CTS affected fibroblasts grown in culture. We noted that CTS (e.g., MBG, ouabain) induced increases in fibroblast collagen production as evidenced by either increased labeled proline incorporation or procollagen expression determined with Western blot. Evidence for Na/K-ATPase signaling (e.g., Src or ERK activation) could be observed as well. Moreover, ROS scavenging or pharmacological or molecular biological Src inhibition prevented increases in proline incorporation and collagen production seen with CTS. An increase in transcription was identified as we saw substantial increases in both mRNA for collagen as well as luciferase in cells transfected with a reporter construct following exposure to CTS. However, we did not see evidence for increased TGF beta signaling in these cells although pharmacological antagonism of the TGF beta system did block CTS stimulated collagen production (51). We next examined how CTS affected Fli-1 expression, stimulated by work performed by Watson and colleagues. Fli-1 is a negative regulator of collagen synthesis (95), and we noted that CTS induce decreases in Fli-1 expression in several types of fibroblasts (cardiac, renal, and dermal). We also observed that decreases in Fli-1 appear to be necessary for MBG to induce increases in collagen. Additional work showed that CTS induce translocation of PKCdelta from the cytosol to the nucleus in a PLC dependent manner. It appears that the translocation of PKCdelta causes Fli-1 phosphorylation and subsequent degradation (94).

These studies next led to work examining the effects of mineralocorticoid antagonists. We should first say that Finotti and colleagues reported 30 years ago that spironolactone and canrenone were antagonists of ouabain binding to the Na/K-ATPase (96). We looked at whether this observation was applicable to our system. *In vitro*, we saw that both spironolactone and canrenone could attenuate MBG-induced increases in collagen production in cardiac fibroblasts. Interestingly, we could not see a substantial effect of aldosterone on cardiac collagen production. Our *in vitro* observations were extended to *in vivo* studies where we saw that administration of spironolactone to rats with experimental renal failure markedly attenuated the observed cardiac fibrosis (94). This suggests the Na/K-ATPase signaling cascade may be a useful target for therapeutic drug development.

Further studies have demonstrated that the effects of MBG (and other CTS) are not specific for cardiac fibroblasts. We have noted that renal fibroblasts have a very similar response as cardiac fibroblasts, suggesting a potential pathological role for MBG in producing renal fibrosis and progressive renal failure. Using MBG infusion in the rat, we saw that such infusion was associated with the induction of Snail, a transcription factor known to be involved in epithelial–mesenchymal transformation (EMT). In LLC-PK1 cells grown in culture, MBG induces EMT in a dose and time dependent way (97).

## Trade-Off with Respect to CTS

**Figure 3:**
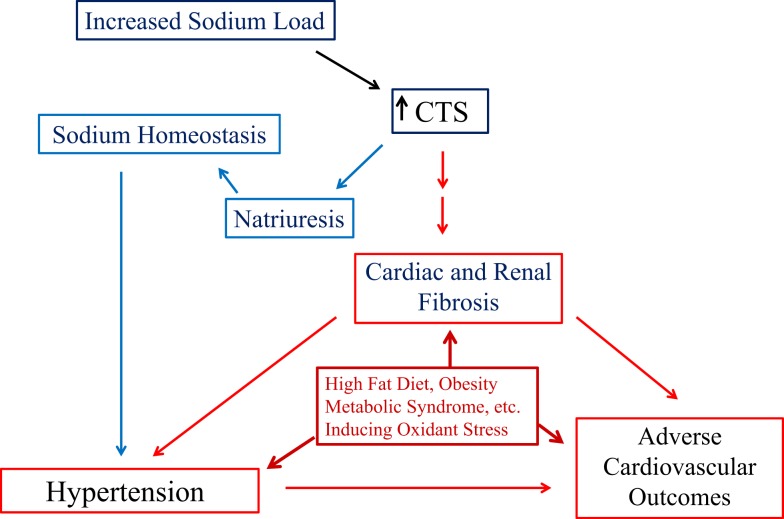
**The schematic shown in figure illustrates the balance between the natriuretic effect of cardiotonic steroids (CTS) and the trade-off of inducing Na/K-ATPase-mediated signal transduction leading to cardiac and renal fibrosis, eventually contributing to the development of hypertension and adverse cardiovascular outcomes**. In addition, chronic metabolic states resulting in the production of reactive oxygen species (ROS) creating oxidative stress may exacerbate the progression of cardiac and renal disease.

With the aforementioned data, we would suggest that the CTS signal cascade through the Na/K-ATPase fits the concept of “trade-off.” Specifically, CTS concentrations increase in response to volume expansion and/or salt loading. These CTS mediate increases in urinary Na excretion, maintaining Na homeostasis, but the endocytosis machinery may fatigue with ongoing stimulation. Moreover, there are other consequences of the elevated CTS concentrations, namely vasoconstriction and hypertension along with fibrosis, which was described above (Figure 3). The fibrosis may lead to further renal insensitivity in terms of natriuresis, and the combination of events cascading to produce progressive cardiovascular disease.

## Future Directions

As we better understand the role of CTS signaling through the Na/K-ATPase, several therapeutic targets come to mind, which may provide novel and effective therapy for different chronic diseases. First, there is the interaction of the CTS with the Na/K-ATPase. This has been addressed experimentally in our laboratory with both active and passive immunization (51, 91, 93, 98) as well as pharmacologically with several different approaches (94, 99). Other groups have developed different substances which can loosely describe as “ouabain antagonists” which we have recently reviewed (5). Rostafuroxin has been very well characterized and appears to have potential for the treatment of hypertension (100, 101). Recently, our laboratory has begun to develop strategies to alter the interaction between the Na/K-ATPase alpha 1 subunit and Src (102). However, it is clear that the aforementioned signaling cascade affords a number of possible sites for intervention including but not limited to the generation of ROS (69), activation of Src and activation of ERK. Unfortunately, these molecular targets will also fit under the general rubric of “trade-off.” Although some aspects of CTS and signaling through the Na/K-ATPase may be maladaptive as we have discussed in this review, it is almost certain that that inhibition of this CTS-Na/K-ATPase pathway may have deleterious effects which need to be navigated.

## Conflict of Interest Statement

Neither Dr. Joe Xie nor Ms. Anna Pearl Shapiro has any conflicts to report. Dr. Joseph Isaac Shapiro currently receives grant support from the NIH concerning this review topic (HL109015 as principal investigator, HL071556 and HL105649 as Co-investigator). Dr. Joseph Isaac Shapiro also holds some awarded patents related to this work (US Patent 8,283,441, Canadian Patents 2641303, 2667251, 2774486, 2360383).

## Abbreviations

Ca, calcium; Cl, chloride; CTS, cardiotonic steroid; EGFR, epidermal growth factor receptor; EMT, epithelial-mesenchymal transformation; ERK, extracellular signal-regulated kinase; FAK, focal adhesion kinase; Fli-1, friend leukemia integration 1 transcription factor; GFR, glomerular filtration rate; Grb2, growth factor receptor-bound protein-2; MBG, marinobufagenin; Na, sodium; Na/K-ATPase, sodium potassium ATPase; NAC, N-acetyl cysteine; NHE3, sodium-hydrogen exchanger 3; PI(3)K, phosphoinositide 3-kinase; PKC, protein kinase C; PLC, phospholipase C; ROS, reactive oxygen species; SERCA, sarcoplasmic endoplasmic reticulum calcium ATPase; Shc, Src homology-2 domain containing protein; SOS, Son of Sevenless protein; TCB, telecinobufagin; TGF, transforming growth factor.
